# *CAT* rs1001179 Single Nucleotide Polymorphism Identifies an Aggressive Clinical Behavior in Chronic Lymphocytic Leukemia

**DOI:** 10.1002/hon.70002

**Published:** 2024-11-14

**Authors:** Marilisa Galasso, Vittoria Salaorni, Riccardo Moia, Valentina Mozzo, Ester Lovato, Chiara Cosentino, Omar Perbellini, Simona Gambino, Ornella Lovato, Maria Elena Carazzolo, Isacco Ferrarini, Francesca M. Quaglia, Massimo Donadelli, Maria G. Romanelli, Carlo Visco, Mauro Krampera, Gianluca Gaidano, Maria T. Scupoli

**Affiliations:** ^1^ Department of Engineering for Innovation Medicine Section of Biomedicine University of Verona Verona Italy; ^2^ Department of Diagnostics and Public Health University of Verona Verona Italy; ^3^ Department of Translational Medicine Division of Hematology University of Piemonte Orientale Novara Italy; ^4^ Veneto Institute of Oncology IOV – IRCCS Padova Italy; ^5^ Hematology Unit Azienda Ospedaliera Universitaria Integrata Verona Verona Italy; ^6^ UOC di Ematologia – Azienda ULSS 8 Berica Vicenza Italy; ^7^ UOSD Diagnostica Genetica e Genomica – Azienda ULSS 8 Berica Vicenza Italy; ^8^ Laboratory of Medical Research University of Verona Verona Italy; ^9^ Department of Medicine University of Verona Verona Italy; ^10^ Department of Neurosciences, Biomedicine and Movement Sciences Section of Biochemistry University of Verona Verona Italy; ^11^ Department of Neurosciences, Biomedicine and Movement Sciences Section Biology and Genetics University of Verona Verona Italy

**Keywords:** *catalase*, chronic lymphocytic leukemia, single nucleotide polymorphism

## Abstract

Chronic lymphocytic leukemia (CLL) is characterized by an extremely variable clinical course. Although several parameters have been shown to be associated with clinical outcomes in patients with CLL, there remains substantial intragroup clinical heterogeneity in otherwise molecularly and staging homogeneous CLL subgroups. We have recently shown that high catalase (CAT) expression identifies patients with an aggressive clinical course and that higher *CAT* expression is associated with the presence of the rs1001179 single nucleotide polymorphism (SNP) T allele in the *CAT* promoter. Herein, we genotyped CLL patients for *CAT* rs1001179 SNP in an exploratory study (*n* = 235) and in a sequential independent validation study (*n* = 531). Time‐to‐event modeling analyses for time‐to‐first‐treatment (TTFT) from the two patients' cohorts showed that TT genotype was associated with a shorter TTFT, independently of other currently used prognostic parameters in CLL. Moreover, the TT genotype identifies CLL patients with a faster clinical progression even within subgroups of patients with low‐risk biological and clinical hallmarks. In conclusion, our data show that the TT genotype identifies CLL patients with a shorter TTFT, pointing to this SNP as a possible prognostic factor, which can improve patients' risk stratification leading to better patient management and personalized therapeutic choices.

## 1 Introduction

Chronic lymphocytic leukemia (CLL), the most prevalent form of leukemia in Western countries, is an incurable disease characterized by an extremely variable clinical course and response to treatment [1, 2, 3]. Over the last decades, several parameters have been shown to be associated with clinical outcomes in patients with CLL. They are used to stratify patients into those with a more indolent course not requiring therapy for a prolonged period versus patients with a more aggressive form of the disease and a reduced time to first treatment (TTFT) [4, 5]. These parameters include Binet staging system, the presence or absence of somatic mutations within the immunoglobulin variable heavy chain genes (*IGHV*), specific chromosomal abnormalities and gene mutations, the expression of the zeta chain of T‐cell receptor associated protein kinase 70 (ZAP‐70), and the expression of the CD38 [6, 7, 8, 9, 10]. However, there remains substantial intragroup clinical heterogeneity in otherwise molecularly and staging homogeneous CLL subgroups [11]. We have recently shown that higher levels of catalase (CAT) identify a more aggressive disease course in CLL whereas lower catalase expression is associated with an indolent clinical behavior [12, 13]. Therefore, differential catalase expression in CLL supports the existence of two main prognostic subtypes, probably because of differences not only in underlying genetic lesions, epigenetic changes, and interactions with the microenvironment, but also in the redox machinery. Lower catalase activity may cause an escalated accumulation of reactive oxygen species (ROS) within leukemic cells, which in turn promotes antitumor signals such as cell death or susceptibility to apoptosis in CLL, thus accounting for a less aggressive behavior of cancer cells [12, 14, 15, 16]. We recently identified genetic and epigenetic mechanisms underlying differential expression of CAT [13]. Specifically, we showed that CLL cells harboring the rs1001179 single nucleotide polymorphism (SNP) T allele within the *CAT* promoter exhibit lower methylation levels and a higher *CAT* expression compared with cells bearing the CC genotype [13].

In this study we investigated the genetic‐based prognostic significance of the *CAT* rs1001179 SNP in CLL. We showed that TT genotype of *CAT* rs1001179 SNP identifies CLL patients with a shorter TTFT and provides prognostic information independently of other currently used prognostic parameters in CLL.

## 2 Material and Methods

### 2.1 Cell Samples

Peripheral blood mononuclear cells (PBMCs) from 235 untreated CLL patients and 123 age‐matched healthy donors (HDs) were collected and cryopreserved at the Hematology Units of the Azienda ULSS 8 Berica, Vicenza (CLL patients, *n* = 172), and the Azienda Ospedaliera Universitaria Integrata (AOUI) of Verona (CLL patients, *n* = 63; HD, *n* = 123). For the validation study, PBMCs from 531 CLL patients were collected and cryopreserved at the Hematology Units of the Azienda Ospedaliero‐Universitaria Maggiore Della Carità of Novara. Clinical annotations at diagnosis are summarized in Tables S1 and S2.

### 2.2 DNA Extraction and Genotyping

Genomic DNA extraction was performed using salting‐out method. Genotyping for *CAT* rs1001179 SNP was assessed by Restriction Fragment Length Polymorphism (RFLP)‐PCR, as previously described [13].

### 2.3 Software and Statistical Analyses

Hardy–Weinberg equilibrium was validated by *χ* ^2^ test. Fisher's exact test, and log‐rank (Mantel‐Cox) test were used as appropriate. Univariate, bivariate and multivariate models for TTFT were generated using Cox proportional hazards regression. TTFT was defined as the interval between CLL diagnosis and date of first treatment or last follow‐up [12]. Differences were considered statistically significant for *p*‐values < 0.05. Graphing and statistical analyses were performed using GraphPad Prism software (v. 7.03, GraphPad Software Inc., CA, USA).

## 3 Results

### 3.1 Exploratory Study

#### 3.1.1 Patients' Characteristics and *CAT* rs1001179 SNP Genotype Distribution

In the exploratory study, we genotyped 235 patients with a median age at the CLL diagnosis of 64 years old and a prevalence of male gender. Data on TTFT were available for more than half of the patients (133 of 235 patients). Of the 132 patients for whom the Binet stage was available, about 70% were at the Binet stage A disease (Table S1).

Distribution of *CAT* rs1001179 SNP genotypes was consistent with the Hardy–Weinberg equilibrium among the 235 CLL patients and the 123 HDs (for CLL patients' rs1001179 polymorphism, *χ* ^2^ = 0.1566, *p* > 0.05; for HDs' rs1001179 polymorphism, *χ* ^2^ = 0.0999, *p* > 0.05). Genotype and allele frequencies among CLL patients and HDs were not significantly different (Table S3). Hence, the *CAT* rs1001179 polymorphism was not associated with CLL risk in the analyzed cohort.

#### 3.1.2 Association With the *CAT* rs1001179 SNP Genetic Variant With Disease Progression

**FIGURE 1:**
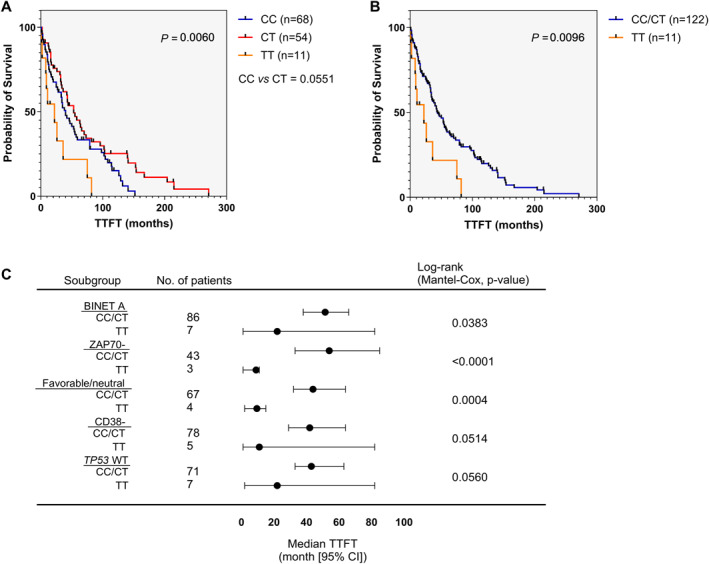
Association between the *CAT* rs1001179 SNP genotypes and disease progression in the exploratory study. Kaplan‐Meier curves of time to first treatment (TTFT) for subgroups of CLL patients distinguished by *CAT* rs1001179 SNP CC (*n* = 68), CT (*n* = 54) and TT (*n* = 11) genotypes (A) and CC/CT (*n* = 122) and TT (*n* = 11) genotypes (B). Forest plot of TTFT (C) for Binet A stage (CC/CT: *n* = 86; TT: *n* = 7), low ZAP70 expression (CC/CT: *n* = 43; TT: *n* = 3), favorable/neutral cytogenetic (CC/CT: *n* = 67; TT: *n* = 4), low CD38 expression (CC/CT: *n* = 78; TT: *n* = 5) and WT *TP53* (CC/CT: *n* = 71; TT: *n* = 7) subgroups of CLL patients distinguished by *CAT* rs1001179 SNP CC/CT or TT genotypes. *p* values are from the log‐rank test.

To investigate the ability of *CAT* rs1001179 SNP to stratify CLL patients, we analyzed the relationship between the SNP genotypes and leukemia progression. We observed significant differences in TTFT between samples bearing CC, CT, or TT genotypes (median TTFT was 40 months for CC‐genotype patients; 53 months for CT‐genotype ones; 22 months for patients bearing TT genotype; *p* = 0.0060; Figure 1A). Moreover, a significant difference in TTFT was observed in Kaplan‐Meier curves between patients bearing the CC/CT genotypes and the mutant TT genotype (median TTFT was 44 months for CC/CT genotype patients while 22 months for patients bearing TT genotype; *p* = 0.0096; Figure 1B). Next, we investigated the association between the *CAT* rs1001179 SNP and TTFT in subsets of patients with early‐stage disease. Forest plot showed significant differences in TTFT between patients bearing the CC/CT genotypes and the mutant TT genotype within the Binet A subset of patients (*p* = 0.0383; Figure 1C). Significant differences were also observed within the subgroup of patients characterized by low ZAP70 expression (*p* < 0.0001; Figure 1C) and among patients with favorable/neutral cytogenetics (*p* = 0.0004; Figure 1C). Furthermore, we documented a trend toward significant association of TT genotype with a shorter TTFT within subgroups of patients characterized by lower CD38 expression (*p* = 0.0514; Figure 1C), and WT *TP*53 (*p* = 0.0560; Figure 1C).

These data show that the TT genotype of *CAT* rs1001179 SNP identifies CLL patients with a poor prognosis within the whole set of patients and among patients with early‐stage disease.

#### 3.1.3 Univariate and Multivariate Analysis of Association of *CAT* rs1001179 SNP With Clinical Progression

We investigated Cox proportional hazard models for TTFT utilizing the *CAT* rs1001179 SNP and the available currently used prognostic parameters. Univariate time‐to‐event analysis identified the rs1001179 TT genotype (LR test *p* = 0.0236, Table 1, Figure 1); increased age at diagnosis (LR test *p* = 0.0377); Binet stage B/C (LR test *p* < 0.0001); unmutated (UM) *IGHV* (LR test *p* = 0.0009); and mutated *TP53* (LR test *p* = 0.0059) as significant predictors of shorter TTFT (Table 1). In a bivariate time‐to‐event analysis, models combining the rs1001179 TT genotype with increased age at diagnosis (LR test *p* = 0.0205); Binet stage B/C (LR test *p* < 0.0001); ZAP70 positivity (LR test *p* = 0.0326); UM‐*IGHV* (LR test *p* = 0.0011); mutated *TP53* (LR test *p* = 0.0050); unfavorable cytogenetics (LR test *p* = 0.0010) were statistically significant (Table S4a). Interestingly, the LR value increased for each prognostic parameter when combined with rs1001179 TT while the statistical significance improved for increased age at diagnosis and mutated *TP53* combined with the TT genotype (Table S4a). Moreover, although ZAP70 positivity and unfavorable cytogenetic were not significantly associated with TTFT in the univariate analysis, models combining each of these parameters with the rs1001179 TT genotype were statistically significant (Table S4a). Remarkably, in multivariate time‐to‐event analysis, a model combining all significant variables identified in the univariate analysis was statistically significant (LR test *p* = 0.0206, Table 1). Next, we investigated Cox proportional hazard models for TTFT utilizing the *CAT* rs1001179 SNP and the available currently used prognostic parameters in an unselected subset of Binet A‐stage patients. Although the rs1001179 TT genotype was not significantly associated with TTFT in a univariate time‐to‐event analysis (Table 2), in a bivariate analysis model combining the rs1001179 TT genotype with unfavorable cytogenetics was statistically significant (LR test *p* = 0.0057; Table S4b). Moreover, the LR value increased for each prognostic parameter when combined with rs1001179 TT (Table S4b). We could not perform multivariate analysis in the Binet A‐stage subgroup of patients due to the lack of some clinical information specifically in those patients bearing the TT genotype. Then, we considered the *CAT* rs1001179 SNP genotypes in relation to biological and clinical parameters. Fisher's test showed no association between the CC/CT or TT genotypes and age, gender, Binet staging, CD38 and ZAP70 expression, *IGHV* and *TP53* mutational status and cytogenetics (Table S5), thus revealing that the *CAT* rs1001179 SNP genotypes are independent of clinical and biological parameters.

**TABLE 1:** Univariate and multivariate models for TTFT in the explorative study.

Variable	*β*s	SE	FP	HR (95% CI)	LR	*p*	Harrell's C
rs1001179 (TT genotype)	0.849	0.337	0.012	2.337 (1.133–4.308)	5.125	**0.0236**	0.532
Age	0.019	0.009	0.037	1.020 (1.001–1039)	4.321	**0.0377**	0.550
Binet B/C	1.220	0.220	< 0.0001	3.389 (2.180–5.182)	26.61	**< 0.0001**	0.627
CD38 positive^a^	0.151	0.243	0.534	1.163 (0.707–1.845)	0.377	0.5394	0.514
ZAP70 positive^b^	−0.070	0.280	0.803	0.933 (0.529–1.594)	0.063	0.8032	0.482
UM‐IGHV^c^	0.701	0.215	0.0011	2.015 (1.330–3.093)	11.05	**0.0009**	0.601
Mutated TP53^d^	1.246	0.398	0.002	3.477 (1.485–7.203)	7.584	**0.0059**	0.547
Cytogenetics (unfavorable)^e^	0.409	0.217	0.059	1.506 (0.977–2.296)	3.443	0.0635	0.551
rs1001179 (TT genotype)	3.112	1.791	0.082	22.46 (0.529–1041)			
Age	0.025	0.038	0.500	1.026 (0.951–1.108)			
Binet B/C	1.050	0.986	0.287	2.857 (0.300–17.13)			
UM‐IGHV^c^	0.348	0.570	0.541	1.417 (0.464–4.546)			
Mutated TP53^d^	2.729	1.247	0.028	15.32 (1.589–344)	13.32	**0.0206**	0.697

*Note:* The significant values were shown in boldface (*p* < 0.05).

Abbreviations: *β*s, beta coefficients; FP, feature‐specific *p*‐value; Harrell's C, concordance index to evaluate the predictive performance of a survival model; LR, likelihood ratio; *p*, global *p*‐value; SE, standard error of estimated coefficients.

^a^ CD38 was determined using a 30% cut‐off.

^b^ ZAP70 was determined using a 20% cut‐off.

^c^ *IGHV* sequencing utilized a 2% cut‐off to discriminate mutated from unmutated *IGHV*.

^d^ *TP53* sequencing utilized a 10% cut‐off to discriminate mutated from wild‐type *TP53*.

^e^ Patients were stratified into major cytogenetic categories, based on NCCN CLL Guidelines: ^3^ favorable (del 13q as a sole aberration), neutral (normal karyotype, trisomy 12q), and unfavorable (11q and/or 17p deletion).

**TABLE 2:** Univariate model for TTFT in Binet A stage patients included in the explorative study.

Variable	*β*s	SE	FP	HR (95% CI)	LR	*p*	Harrell's C
rs1001179 (TT genotype)	0.870	0.433	0.044	2.387 (0.915–5.146)	3.234	0.0721	0.530
Age	0.032	0.012	0.006	1.032 (1.009–1.057)	7.505	**0.006**	0.612
CD38 positive^a^	0.068	0.307	0.824	1.071 (0.564–1.904)	0.049	0.825	0.517
ZAP70 positive^b^	−0.172	0.325	0.596	0.841 (0.433–1.569)	0.286	0.593	0.497
UM‐IGHV^c^	0.851	0.262	0.0011	2.342 (1.411–3.955)	10.86	**0.0010**	0.636
Mutated TP53^d^	1.797	0.478	0.0002	6.029 (2.160–14.57)	10.17	**0.0014**	0.577
Cytogenetics (unfavorable)^e^	0.439	0.272	0.106	1.552 (0.899–2.630)	2.522	0.1123	0.553

*Note:* The significant values were shown in boldface (*p* < 0.05).

Abbreviations: *β*s, beta coefficients; FP, feature‐specific *p*‐value; Harrell's C, concordance index to evaluate the predictive performance of a survival model; LR, likelihood ratio; *p*, global *p*‐value; SE, standard error of estimated coefficients.

^a^ CD38 was determined using a 30% cut‐off.

^b^ ZAP70 was determined using a 20% cut‐off.

^c^ *IGHV* sequencing utilized a 2% cut‐off to discriminate mutated from unmutated *IGHV*.

^d^ *TP53* sequencing utilized a 10% cut‐off to discriminate mutated from wild‐type *TP53*.

^e^ Patients were stratified into major cytogenetic categories, based on NCCN CLL Guidelines: ^3^ favorable (del 13q as a sole aberration), neutral (normal karyotype, trisomy 12q), and unfavorable (11q and/or 17p deletion).

Taken together, these data identify the rs1001179 TT genotype as a significant predictor of disease progression that provides complementary information to the currently used CLL prognostic parameters.

### 3.2 Validation Study

#### 3.2.1 Patients' Characteristics and *CAT* rs1001179 SNP Genotype Distribution

In the validation study, the median age at the CLL diagnosis was 70 years old with a predominance of male gender. TTFT data were available for 464 CLL patients of the 531 (87%) analyzed for CAT rs1001179 SNP. Among the 506 patients with available Binet stage information, approximately 82% were diagnosed with Binet A disease (Table S2). Consistently with results from the exploratory study, genotype frequencies among patients from the validation study were 55.9% for CC, 38% for CT and 6.1% for TT whilst the allele frequencies were 75% for the major C allele and 25% for the minor T allele.

#### 3.2.2 Association With the *CAT* rs1001179 SNP Genetic Variant With Disease Progression

**FIGURE 2:**
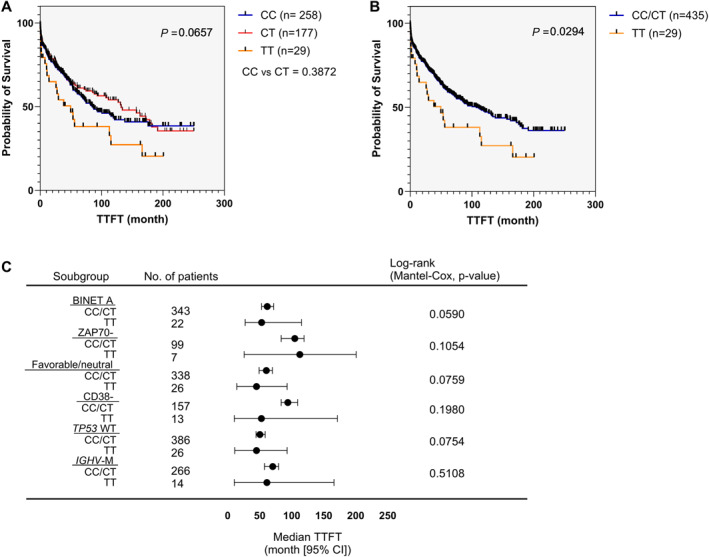
Association between the *CAT* rs1001179 SNP genotypes and CLL progression‐validation study. Kaplan‐Meier curves of time to first treatment (TTFT) for subgroups of CLL patients distinguished by *CAT* rs1001179 SNP CC (*n* = 258), CT (*n* = 177) and TT (*n* = 29) genotypes (A) and CC/CT (*n* = 435) and TT (*n* = 29) genotypes (B). Forest plot of TTFT (C) for Binet A stage (CC/CT: *n* = 343; TT: *n* = 22), low ZAP70 expression (CC/CT: *n* = 99; TT: *n* = 7), favorable/neutral cytogenetic (CC/CT: *n* = 338; TT: *n* = 26), low CD38 expression (CC/CT: *n* = 157; TT: *n* = 13), WT *TP53* (CC/CT: *n* = 386; TT: *n* = 26), M‐*IGHV* (CC/CT: *n* = 266; TT: *n* = 14) subgroups of CLL patients distinguished by *CAT* rs1001179 SNP CC/CT or TT genotypes. *p* values are from the log‐rank test.

In the validation cohort, although we observed no significant differences in TTFT between samples bearing CC, CT, or TT genotypes (median TTFT was 84.7 months for CC‐genotype patients; 132 months for those with CT genotype; 49.7 months for TT‐genotype patients; *p* = 0.0657; Figure 2A), we confirmed a significant difference in TTFT between patients bearing the CC/CT genotypes and the mutant TT genotype (median TTFT was 107.1 months for patients with CC/CT genotypes while 49.7 months for TT‐genotype patients; *p* = 0.0294; Figure 2B). Moreover, in patients with indolent disease, we documented a trend toward significant association of TT genotype with a more aggressive disease within subgroups of patients characterized by Binet A stage (*p* = 0.0590; Figure 2C); low ZAP70 expression (*p* = 0.1054; Figure 2C); favorable/neutral cytogenetics (*p* = 0.0759; Figure 2C); lower CD38 expression (*p* = 0.1980; Figure 2C); and WT *TP*53 (*p* = 0.0754; Figure 2C). We documented no significant differences in TTFT between patients bearing the CC/CT genotypes and the mutant TT genotype in the subgroup of patients characterized by somatic mutations within the immunoglobulin variable heavy chain genes (M‐*IGHV*) (*p* = 0.5108; Figure 2C).

#### 3.2.3 Univariate and Multivariate Analysis of Association of *CAT* rs1001179 SNP With Clinical Progression

Univariate time‐to‐event analysis identified the rs1001179 TT genotype (LR test *p* = 0.044, Table 3; Figure 2); Binet stage B/C, CD38 positivity, UM‐*IGHV*, mutated *TP53*, and unfavorable cytogenetic (LR test *p* < 0.0001); ZAP70 positivity (LR test *p* = 0.0005) as significant predictors of shorter TTFT (Table 3). In bivariate time‐to‐event analysis, models combining the rs1001179 TT genotype with Binet stage B/C, CD38 positivity, UM‐*IGHV*, mutated *TP53*, or unfavorable cytogenetics were statistically significant (LR test *p* < 0.0001 for each model; Table S6a). Also, the rs1001179 TT genotype combined with ZAP70 positivity was statistically significant (LR test *p* = 0.0011; Table S6a). Interestingly, as for exploratory study the LR value increased for each prognostic parameter when combined with rs1001179 TT (Table S6a). Moreover, increased age at diagnosis reached the statistical significance when combined with the TT genotype (LR test *p* = 0.057; Table S6a). Remarkably, as in the exploratory study, in multivariate time‐to‐event analysis, a model combining all significant variables identified in the univariate analysis was statistically significant (LR test *p* = < 0.0001, Table 3). Next, we tested Cox proportional hazard models for TTFT combining the *CAT* rs1001179 SNP and the available currently used prognostic parameters in a subset of Binet A‐stage patients included in the validation study (Table 4). Consistently with the explorative study, the rs1001179 TT genotype was not significantly associated with TTFT in univariate time‐to‐event analysis. Moreover, although the LR value increased for each prognostic parameter when combined with rs1001179 TT, the statistical significance of association between TTFT and each prognostic parameters did not significantly differ from the univariate analysis (Table S6b). Remarkably, in multivariate time‐to‐event analysis within the subset of Binet A‐stage patients, a model combining all significant variables identified in the univariate analysis was statistically significant (LR test *p* < 0.0001, Table 4). Then, we considered the *CAT* rs1001179 SNP genotypes in relation to biological and clinical parameters in the validation cohort. Consistently with the exploratory study, we documented no association between the CC/CT or TT genotypes and age, gender, Binet staging, CD38 and ZAP70 expression, *IGHV* and *TP53* mutational status or cytogenetics (Table S7).

**TABLE 3:** Univariate and multivariate models for TTFT in the validation study.

Variable	*β*s	SE	FP	HR (95% CI)	LR	*p*	Harrell's C
rs1001179 (TT genotype)	0.505	0.235	0.031	1.658 (1.014–2.556)	4.052	**0.044**	0.518
Age	0.008	0.006	0.188	1.009 (0.995–1.022)	1.748	0.186	0.537
Binet B/C	2.036	0.154	< 0.0001	7.661 (5.635–10.32)	131.9	**< 0.0001**	0.672
CD38 positive^a^	1.021	0.190	< 0.0001	2.775 (1.898–4.012)	25.60	**< 0.0001**	0.612
ZAP70 positive^b^	0.639	0.186	0.0006	1.895 (1.321–2.746)	12.16	**0.0005**	0.59
UM‐IGHV^c^	−1.366	0.140	< 0.0001	0.255 (0.193–0.335)	93.81	**< 0.0001**	0.665
Mutated TP53^d^	0.975	0.201	< 0.0001	2.652 (1.752–3.869)	18.63	**< 0.0001**	0.549
Cytogenetics (unfavorable)^e^	1.041	0.172	< 0.0001	2.833 (1.996–3.933)	29.52	**< 0.0001**	0.568
rs1001179 (TT genotype)	0.406	0.310	0.190	1.501 (0.775–2.651)			
Binet B/C	1.953	0.238	< 0.0001	7.053 (4.367–11.14)			
CD38 positive^a^	0.538	0.220	0.014	1.713 (1.106–2.625)			
ZAP70 positive^b^	0.194	0.210	0.357	1.214 (0.805–1.843)			
UM‐IGHV^c^	1.136	0.239	< 0.0001	3.116 (1.949–4.981)			
Mutated TP53^d^	1.263	0.327	0.0001	3.536 (1.814–6.579)			
Cytogenetics (unfavorable)^e^	−0.1789	0.304	0.556	0.836 (0.451–1.494)	133.7	**< 0.0001**	0.773

*Note:* The significant values were shown in boldface (*p* < 0.05).

Abbreviations: *β*s, beta coefficients; FP, feature‐specific *p*‐value; Harrell's C, concordance index to evaluate the predictive performance of a survival model; LR, likelihood ratio; *p*, global *p*‐value; SE, standard error of estimated coefficients.

^a^ CD38 was determined using a 30% cut‐off.

^b^ ZAP70 was determined using a 11% cut‐off.

^c^ *IGHV* sequencing utilized a 2% cut‐off to discriminate mutated from unmutated *IGHV*.

^d^ *TP53* sequencing utilized a 10% cut‐off to discriminate mutated from wild‐type *TP53*.

^e^ Patients were stratified into major cytogenetic categories, based on NCCN CLL Guidelines: favorable (del 13q as a sole aberration), neutral (normal karyotype, trisomy 12q), and unfavorable (11q and/or 17p deletion).

**TABLE 4:** Univariate and multivariate models for TTFT in Binet A stage patients included in the validation study.

Variable	*β*s	SE	FP	HR (95% CI)	LR	*p*	Harrell's C
rs1001179 (TT genotype)	0.544	0.291	0.062	1.723 (0.926–2.934)	3.011	0.082	0.519
Age	0.011	0.008	0.207	1.011 (0.994–1.028)	1.608	0.204	0.548
CD38 positive^a^	0.778	0.238	0.001	2.179 (1.342–3.433)	9.431	**0.002**	0.573
ZAP70 positive^b^	0.485	0.215	0.024	1.624 (1.066–2.491)	5.100	**0.023**	0.578
UM‐IGHV^c^	1.329	0.176	< 0.0001	3.778 (2.671–5.343)	53.35	**< 0.0001**	0.652
Mutated TP53^d^	0.858	0.293	0.003	2.360 (1.266–4.033)	6.871	**0.008**	0.534
Cytogenetics (unfavorable)^e^	1.034	0.234	< 0.0001	2.813 (1.731–4.364)	15.40	**< 0.0001**	0.556
rs1001179 (TT genotype)	0.502	0.361	0.1644	1.652 (0.759–3.183)			
CD38 positive^a^	0.580	0.256	0.023	1.787 (1.065–2.918)			
ZAP70 positive^b^	0.102	0.235	0.661	1.108 (0.699–1.764)			
UM‐IGHV^c^	1.246	0.263	< 0.0001	3.477 (2.070–5.819)			
Mutated TP53^d^	1.031	0.403	0.0105	2.805 (1.202–5.920)			
Cytogenetics (unfavorable)^e^	−0.043	0.354	0.902	0.957 (0.463–1.874)	46.54	**< 0.0001**	0.692

*Note:* The significant values were shown in boldface (*p* < 0.05).

Abbreviations: *β*s, beta coefficients; SE, standard error of estimated coefficients; FP, feature‐specific *p*‐value; LR, likelihood ratio; *p*, global *p*‐value; Harrell's C, concordance index to evaluate the predictive performance of a survival model.

^a^ CD38 was determined using a 30% cut‐off.

^b^ ZAP70 was determined using a 11% cut‐off.

^c^ *IGHV* sequencing utilized a 2% cut‐off to discriminate mutated from unmutated *IGHV*.

^d^ *TP53* sequencing utilized a 10% cut‐off to discriminate mutated from wild‐type *TP53*.

^e^ Patients were stratified into major cytogenetic categories, based on NCCN CLL Guidelines: ^3^ favorable (del 13q as a sole aberration), neutral (normal karyotype, trisomy 12q), and unfavorable (11q and/or 17p deletion).

## 4 Discussion

In this study, we show that the *CAT* rs1001179 SNP within the *catalase* promoter is of clinical relevance in CLL since it is associated with more aggressive disease in both the exploratory and the independent validation study. Although several biological parameters enable the classification of CLL patients into risk groups, predicting which patients will progress remains a significant clinical challenge [11]. We propose that the *CAT* rs1001179 SNP could provide information independent from standard prognostic markers in predicting disease progression risk in CLL.

The *CAT* rs1001179 SNP is a common functional polymorphism within the promoter of the *CAT* gene, substituting allele C with T at position 330 relative to the ATG on chromosome 11 [16, 17]. Herein, we show that the TT genotype of *CAT* rs1001179 SNP is a significant predictor of a more aggressive clinical behavior in CLL; in contrast, the CC and CT genotypes are associated with a more indolent disease course. Remarkably, time‐to‐event models for TTFT show that the *CAT* rs1001179 SNP provides independent information of currently used prognostic parameters, pointing to this SNP as a relevant genetic marker that can improve the currently used prognostic tests.

The TT genotype identifies CLL patients with a faster clinical progression even within subgroups of patients with low‐risk biological and clinical hallmarks, highlighting the prognostic significance of the TT genotype also in these subsets of patients, who will mostly benefit from an improved prognostic system. Indeed, although many patients with favorable prognostic markers never progress to the point of therapeutic intervention, others experience a more accelerated course and require therapy [11]. The accurate identification of these patients in the initial state of the disease would strongly improve their clinical management.

In agreement with our data, the *CAT* rs1001179 SNP has been proposed as a prognostic factor in ovarian cancer patients, resulting the CT and TT genotypes associated with poor survival [18]. Furthermore, this polymorphism has been shown to be associated with the incidence risk to develop prostate cancer, hepatocellular carcinoma, skin cancer, and cervical cancer [19, 20, 21, 22]. A recent comprehensive metanalysis also revealed a significant correlation of *CAT* rs1001179 SNP with susceptibility to blood‐ and bone‐marrow‐related cancers, skin cancers, gastrointestinal‐tract‐related cancers, prostate cancer, and gynecologic cancers [23]. Herein, we report that CLL and HD samples show comparable genotypes and allele frequencies, thus excluding the association between the *CAT* rs1001179 SNP and susceptibility to CLL risk in the analyzed cohort. Our finding is in line with previous results showing that the *CAT* rs11001179 is not a risk factor for non‐Hodgkin lymphoma development [24]. Consistently, previously large scale genome‐wide association studies (GWAS) did not identify the *CAT* rs1001179 as a SNP for CLL risk [25, 26, 27, 28, 29, 30]. Taken together, these data suggest that the impact of this SNP on cancer risk may be influenced by specific cancer‐cell contexts.

The *CAT* rs1001179 SNP has been associated with higher levels of CAT in healthy blood cells [17]. In line with this finding, we have recently shown that CLL cells harboring the rs1001179 SNP T allele exhibit significantly higher CAT expression levels compared with cells bearing the CC genotype [13]. Catalase is a ubiquitous antioxidant enzyme that works cooperatively with other antioxidant enzymes to protect cells from an excess of ROS derived from endogenous metabolism or external microenvironment [16]. The expression of catalase and other antioxidant enzymes is often altered in cancer, resulting in aberrant levels of ROS, and catalase as well as other antioxidant enzymes play an important dichotomous role in cancer [16]. Specifically, while catalase can protect cells from tumor initiation and progression [31, 32, 33, 34] due to its role in preventing the accumulation of dangerous levels of oxidants, many cancer cells require high oxidant detoxifying systems and upregulation of catalase for tumor progression and metastasis to compensate for high ROS production and prevent the action of cell death processes [15, 35, 36, 37, 38]. Consistently with the catalase protumor role, we have recently documented that higher levels of catalase and decreased levels of cellular ROS are associated with a faster progression of CLL [12, 13]. Therefore, the mechanistic explanation of the finding that the *CAT* rs1001179 SNP is a determinant of a dismal outcome in CLL could rely on the antioxidant role of catalase in regulating ROS and ROS‐mediated cell death. Elevated catalase levels, decreasing ROS cellular levels, could reduce antitumor signals in leukemic cells, that is, cell death or susceptibility to apoptosis, thereby influencing disease progression [16]. Although this hypothesis deserves further investigation to be validated, our study could lead to the development of new therapeutic strategies targeting redox pathways that could implement the effectiveness of current therapies and overcome drug resistance in CLL.

In conclusion, our data show for the first time that the TT genotype of CAT rs1001179 SNP identifies CLL patients with a shorter TTFT, pointing to this genetic polymorphism as a possible prognostic factor in CLL. In addition, the rs1001179 SNP can improve risk stratification of patients with early‐stage disease, which can lead to better patient management and personalized therapeutic choices.

## Author Contributions

M.G. designed the study, performed experiments, analyzed and interpreted data, wrote the manuscript; V.S. performed experiments, analyzed and interpreted data; V.M., E. L. and C. C. managed clinical data and interpreted data; S.G., O.L. and M.E.C. performed experiments; F.M.Q and M.K. managed clinical data; I.F., O.P., M.D., M.G.R. and C.V. contributed to study design and interpreted data; R.M. and G.G. interpreted data; M.T.S. designed and coordinated the study, interpreted data, and wrote the manuscript. All authors reviewed the manuscript.

## Ethics Statement

The study was conducted in accordance with the Declaration of Helsinki, and the protocol was approved by the respective local Ethics Committees.

## Conflicts of Interest

The authors declare no conflicts of interest.

### Peer Review

The peer review history for this article is available at https://www.webofscience.com/api/gateway/wos/peer-review/10.1002/hon.70002.

## Supporting information

Supporting Information S1

## Data Availability

The data that supports the findings of this study are available in the main text and in the supplementary material of this article.
