# Supplementary material for: CAT rs1001179 Single Nucleotide Polymorphism Identifies an Aggressive Clinical Behavior in Chronic Lymphocytic Leukemia
Source: Hematol Oncol. 2024 Nov 14;42(6):e70002. doi: 10.1002/hon.70002 (PMC11590045; doi:10.1002/hon.70002)
Supplement: Supplementary file 1 — Supporting Information S1 [file HON-42-e70002-s001.docx]

***CAT* rs1001179 single nucleotide polymorphism identifies an aggressive clinical behavior in chronic lymphocytic leukemia**

Marilisa Galasso^1^, Vittoria Salaorni^2^, Riccardo Moia^3^, Valentina Mozzo^4^, Ester Lovato^1,5^, Chiara Cosentino^3^, Omar Perbellini^6,7^, Simona Gambino^1^, Ornella Lovato^8^, Maria Elena Carazzolo^9^, Isacco Ferrarini^1,5^, Francesca M. Quaglia^5^, Massimo Donadelli^10^, Maria G. Romanelli^11^, Carlo Visco^1,5^ Mauro Krampera^1,5^, Gianluca Gaidano^3^, Maria T. Scupoli^1,8^

*^1^Department of Engineering for Innovation Medicine, Section of Biomedicine, University of Verona, Italy.*

*^2^Department of Diagnostics and Public Health, University of Verona, Italy.*

*^3^Department of Translational Medicine, Division of Hematology, University of Piemonte Orientale,* *Novara, Italy.*

*^4^Veneto Institute of Oncology IOV – IRCCS, Padova, Italy.*

*^5^Hematology Unit, Azienda Ospedaliera Universitaria Integrata Verona, Verona, Italy.*

*^6^UOC di Ematologia - Azienda ULSS 8 Berica, Vicenza, Italy.Research Center LURM, Interdepartmental*

*^7^UOSD Diagnostica Genetica e Genomica - Azienda ULSS 8 Berica, Vicenza, Italy.*

*^8^ Laboratory of Medical Research, University of Verona, Verona, Italy.*

*^9^Department of Medicine, University of Verona, Verona, Italy.*

*^10^Department of Neurosciences, Biomedicine and Movement Sciences, Section of Biochemistry, University of Verona, Verona, Italy.*

*^11^Department of Neurosciences, Biomedicine and Movement Sciences, Section Biology and Genetics, University of Verona, Verona, Italy.*

**Tables**

**Table S1: Clinical and biological characteristics of CLL patients included in the explorative study**

|  | n=235 |
| --- | --- |
| *Gender*  Male  Female | 147 (62.6%)  88 (37.4%) |
| *Age at the diagnosis (years)*  Median (range) | 64 (36-92) |
| *^a^TTFT (months)*  Median (range) | 36 (1-271) |
| *Binet*  Binet A  Binet B  Binet C  NA | 93 (39.6%)  28 (11.9%)  11 (4.7%)  103 (43.8%) |
| *^b^CD38*  Negative  Positive  NA | 85 (36.2%)  27 (11.5%)  123 (52.3%) |
| *^c^ZAP70*  Negative  Positive  NA | 47 (20.0%)  25 (10.6%)  163 (69.4%) |
| *^d^IGHV*  UM  M  NA | 94 (40.0%)  68 (28.9%)  73 (31.1%) |
| *^e^TP53*  WT  MUT  NA | 162 (68.9.%)  24 (10.2%)  49 (20.9%) |
| ^f^*Cytogenetics*  Favorable  Neutral  Unfavorable  NA | 39 (16.6%)  33 (14.0%)  45 (19.1%)  118 (50.2%) |

^a^TTFT: time-to-first-treatment;

^b^CD38 was determined using a 30% cut-off;

^c^ZAP70 was determined using a 20% cut-off;

^d^*IGHV* sequencing utilized a 2% cut-off to discriminate mutated from unmutated *IGHV*; M: mutated; UM: unmutated;

^e^*TP53* sequencing utilized a 10% cut-off to discriminate mutated from wild-type *TP53*; MUT: mutated; WT: wild-type;

^f^Patients were stratified into major cytogenetic categories, based on NCCN CLL Guidelines: ^1^ favorable (del 13q as a sole aberration), neutral (normal karyotype, trisomy 12q), and unfavorable (11q and/or 17p deletion);

NA: not available.

**Table S2: Clinical and biological characteristics of CLL patients included in the validation study**

|  | n=531 |
| --- | --- |
| *Gender*  Male  Female | 315 (59.3%)  216 (40.7%) |
| *Age at the diagnosis (years)*  Median (range) | 70 (39-92) |
| *^a^TTFT (months)*  Median (range) | 46.9 (1-250) |
| *Binet*  Binet A  Binet B  Binet C  NA | 413 (77.8%)  52 (9.8%)  41 (7.7%)  25 (4.7%) |
| *^b^CD38*  Negative  Positive  NA | 215 (40.5%)  73 (13.7%)  243 (45.8%) |
| *^c^ZAP70*  Negative  Positive  NA | 130 (24.5%)  154 (29%)  247 (46.5%) |
| *^d^IGHV*  UM  M  NA | 196 (36.9%)  321 (60.5%)  14 (2.6%) |
| *^e^TP53*  WT  MUT  NA | 470 (88.5%)  50 (9.4%)  11 (2.1%) |
| ^f^*Cytogenetics*  Favorable  Neutral  Unfavorable  NA | 192 (36.2%)  224 (42.2%)  71 (13.4%)  44 (8.2%) |

^a^TTFT: time-to-first-treatment;

^b^CD38 was determined using a 30% cut-off;

^c^ZAP70 was determined using a 11% cut-off;

^d^*IGHV* sequencing utilized a 2% cut-off to discriminate mutated from unmutated *IGHV*; M: mutated; UM: unmutated;

^e^*TP53* sequencing utilized a 10% cut-off to discriminate mutated from wild-type *TP53*; MUT: mutated; WT: wild-type;

^f^Patients were stratified into major cytogenetic categories, based on NCCN CLL Guidelines^1^: favorable (del 13q as a sole aberration), neutral (normal karyotype, trisomy 12q), and unfavorable (11q and/or 17p deletion);

NA: not available.

**Table S3: Distributions of *CAT* rs1001179 SNP genotypes in CLL patients and HD**

| *CAT* rs1001179 | CLL (%) | HD (%) | OR | 95% CI | *P* | |
| --- | --- | --- | --- | --- | --- | --- |
| CC | 130 (55.3) | 68 (55.3) | 1.0 | - | | - |
| CT | 88 (37.4) | 46 (37.4) | 1.00 | 0.627 – 1.573 | | *>0,9999* |
| TT | 17 (7.3) | 9 (7.3) | 0.99 | 0.439 – 2.472 | | *>0,9999* |
| CT+TT | 105 | 55 | 0.99 | 0.637 – 1.536 | | *>0,9999* |
|  |  |  |  |  | |  |
| C | 348 (74) | 182 (74) | 1.0 | - | | *-* |
| T | 122 (26) | 64 (26) | 0.99 | 0.705 – 1.413 | | *>0,9999* |

OD: odd ratio; CI: confidence interval; ns: not significant

| Variable | βs | SE | *FP* | HR (95% CI) | LR | *P* | Harrell's C |
| --- | --- | --- | --- | --- | --- | --- | --- |
| *rs1001179*  *(TT genotype)*  *Age* | 0.702  0.015 | 0.349  0.009 | 0.044  0.103 | 2.018 (0.959 – 3.820)  1.016 (0.997 – 1.035) | 7.778 | **0.0205** | **0.590** |
| *rs1001179*  *(TT genotype)*  *Binet B/C* | 0.695  1.201 | 0.339  0.221 | 0.0403  <0.0001 | 2.003 (0.968 – 3.706)  3.324 (2.134 – 5.095) | 30.15 | **<0.0001** | 0.643 |
| *rs1001179*  *(TT genotype)*  *^b^CD38 positive* | 0.719  0.093 | 0.381  0.246 | 0.059  0.707 | 2.052 (0.899 – 4.080)  1.097 (0.663 – 1.751) | 3.369 | 0.1855 | 0.540 |
| *rs1001179*  *(TT genotype)*  *^c^ZAP70 positive* | 1.780  -0.164 | 0.576  0.285 | 0.002  0.565 | 5.930 (1.658 – 16.85)  0.849 (0.477 – 1.464) | 6.845 | **0.0326** | 0.519 |
| *rs1001179*  *(TT genotype)*  *^d^UM-IGHV* | 0.697  0.688 | 0.399  0.215 | 0.0804  0.0014 | 2.008 (0.836 – 4.090)  1.989 (1.312 – 3.056) | 13.60 | **0.0011** | 0.613 |
| *rs1001179*  *(TT genotype)*  *^e^mutated TP53* | 0.773  1.324 | 0.406  0.401 | 0.0571  0.0010 | 2.167 (0.892 – 4.499)  3.760 (1.597 – 7.857) | 10.58 | **0.0050** | 0.574 |
| *rs1001179*  *(TT genotype*  *^f^Cytogenetics*  *(unfavorable)* | 2.002  0.442 | 0.506  0.217 | <0.0001  0.0425 | 7.400 (2.438 – 18.54)  1.555 (1.008 – 2.373) | 13.85 | **0.0010** | 0.584 |

**Table S4a: Bivariate models for TTFT in the explorative study**

^b^CD38 was determined using a 30% cut-off;

^c^ZAP70 was determined using a 20% cut-off;

^d^*IGHV* sequencing utilized a 2% cut-off to discriminate mutated from unmutated *IGHV*;

^e^*TP53* sequencing utilized a 10% cut-off to discriminate mutated from wild-type *TP53*;

^f^Patients were stratified into major cytogenetic categories, based on NCCN CLL Guidelines: ^3^ favorable (del 13q as a sole aberration), neutral (normal karyotype, trisomy 12q), and unfavorable (11q and/or 17p deletion);

βs: beta coefficients; SE: standard error of estimated coefficients; FP: feature-specific p-value; LR: likelihood ratio; P: global p-value; Harrell's C: concordance index to evaluate the predictive performance of a survival model. The significant values were shown in boldface (P < 0.05).

**Table S4b: Bivariate models for TTFT in Binet A stage patients included in the explorative study**

| Variable | βs | SE | *FP* | HR (95% CI) | LR | *P* | Harrell's C |
| --- | --- | --- | --- | --- | --- | --- | --- |
| *rs1001179*  *(TT genotype)*  *Age* | 0.637  0.029 | 0.441  0.012 | 0.148  0.014 | 1.892 (0.716 – 4.163)  1.030 (1.006 – 1.054) | 9.299 | **0.0096** | 0.6353 |
| *rs1001179*  *(TT genotype)*  *^b^CD38 positive* | 0.836  -0.033 | 0.489  0.317 | 0.087  0.918 | 2.307 (0.778 – 5.508)  0.967 (0.501 – 1.752) | 2.455 | 0.293 | 0.5234 |
| *rs1001179*  *(TT genotype)*  *^c^ZAP70 positive* | 1.914  -0.295 | 0.809  0.339 | 0.018  0.384 | 6.780 (0.997 – 28.16)  0.744 (0.371 – 1.420) | 4.120 | 0.1274 | 0.5277 |
| *rs1001179*  *(TT genotype)*  *^d^UM-IGHV* | 0.701  0.832 | 0.476  0.263 | 0.1408  0.0015 | 2.015 (0.693 – 4.660)  2.298 (1.381 – 3.886) | 12.68 | **0.0018** | 0.6475 |
| *rs1001179*  *(TT genotype)*  *^e^mutated TP53* | 0.788  1.878 | 0.487  0.483 | 0.1055  0.0001 | 2.200 (0.745 – 5.242)  6.539 (2.326 – 15.98) | 12.35 | **0.0021** | 0.609 |
| *rs1001179*  *(TT genotype*  *^f^Cytogenetics*  *(unfavorable)* | 3.084  0.489 | 0.842  0.274 | 0.0002  0.0747 | 21.85 (3.090 – 100.1)  1.631 (0.941 – 2.778) | 10.34 | **0.0057** | 0.577 |

^b^CD38 was determined using a 30% cut-off;

^c^ZAP70 was determined using a 20% cut-off;

^d^*IGHV* sequencing utilized a 2% cut-off to discriminate mutated from unmutated *IGHV*;

^e^*TP53* sequencing utilized a 10% cut-off to discriminate mutated from wild-type *TP53*;

^f^Patients were stratified into major cytogenetic categories, based on NCCN CLL Guidelines: ^3^ favorable (del 13q as a sole aberration), neutral (normal karyotype, trisomy 12q), and unfavorable (11q and/or 17p deletion);

**Table S5: Association between *CAT* rs1001179 SNP genotypes and clinical parameters in the explorative study**

| Parameters | CC/CT | TT | *P* |
| --- | --- | --- | --- |
| *Gender*  Male  Female | 136  82 | 11  6 | *>0.099* |
| *Binet*  Binet A  Binet B/C | 86  35 | 7  4 | *0.731* |
| *^$^CD38*  Negative  Positive | 80  23 | 5  4 | *0.215* |
| †ZAP70  Negative  Positive | 44  23 | 3  2 | *>0.099* |
| *^‡^IGHV*  UM  M | 87  65 | 7  3 | *0.522* |
| *^¥^TP53*  WT  MUT | 150  24 | 12  0 | 0.369 |
| ^§§^*Cytogenetics*  Favorable/ Neutral  Unfavorable | 68  43 | 4  2 | *>0.099* |

^b^CD38 was determined using a 30% cut-off;

^c^ZAP70 was determined using a 20% cut-off;

^d^*IGHV* sequencing utilized a 2% cut-off to discriminate mutated from unmutated *IGHV*; M: mutated; UM: unmutated;

^e^*TP53* sequencing utilized a 10% cut-off to discriminate mutated from wild-type *TP53*; MUT: mutated; WT: wild-type;

^f^Patients were stratified into major cytogenetic categories, based on NCCN CLL Guidelines: ^1^ favorable (del 13q as a sole aberration), neutral (normal karyotype, trisomy 12q), and unfavorable (11q and/or 17p deletion).

| Variable | βs | SE | *FP* | HR (95% CI) | LR | *P* | Harrell's C |
| --- | --- | --- | --- | --- | --- | --- | --- |
| *rs1001179*  *(TT genotype)*  *Age* | 0.500  0.008 | 0.234  0.006 | 0.033  0.198 | 1.649 (1.009 – 2.543)  1.008 (0.995 – 1.022) | 5.719 | **0.057** | 0.545 |
| *rs1001179*  *(TT genotype)*  *Binet B/C* | 0.574  2.053 | 0.247  0.154 | 0.020  <0.0001 | 1.775 (1.055 – 2.801)  7.794 (5.727 – 10.51) | 136.5 | **<0.0001** | 0.683 |
| *rs1001179*  *(TT genotype)*  *^b^CD38 positive* | 0.234  1.027 | 0.304  0.190 | 0.441  <0.0001 | 1.264 (0.659 – 2.202)  2.792 (1.908 – 4.039) | 26.15 | **<0.0001** | 0.618 |
| *rs1001179*  *(TT genotype)*  *^c^ZAP70 positive* | 0.398  0.639 | 0.304  0.186 | 0.190  0.0006 | 1.490 (0.778 – 2.593)  1.895 (1.320 – 2.745) | 13.70 | **0.0011** | 0.604 |
| *rs1001179*  *(TT genotype)*  *^d^UM-IGHV* | 0.426  -1.361 | 0.234  0.140 | 0.069  <0.0001 | 1.532 (0.936 – 2.364)  0.256 (0.194 – 0.337) | 96.75 | **<0.0001** | 0.670 |
| *rs1001179*  *(TT genotype)*  *^e^mutated TP53* | 0.518  0.988 | 0.234  0.201 | 0.027  <0.0001 | 1.680 (1.027 – 2.593)  2.687 (1.775 – 3.923) | 22.88 | **<0.0001** | 0.561 |
| *rs1001179*  *(TT genotype*  *^f^Cytogenetics*  *(unfavorable)* | 0.558  1.077 | 0.236  0.173 | 0.018  <0.0001 | 1.749 (1.067 – 2.708)  2.937 (2.066 – 4.088) | 34.35 | **<0.0001** | 0.580 |

**Table S6a: Bivariate models for TTFT in the validation study**

^b^CD38 was determined using a 30% cut-off;

^c^ZAP70 was determined using a 11% cut-off;

^d^*IGHV* sequencing utilized a 2% cut-off to discriminate mutated from unmutated *IGHV*;

^e^*TP53* sequencing utilized a 10% cut-off to discriminate mutated from wild-type *TP53*;

^f^Patients were stratified into major cytogenetic categories, based on NCCN CLL Guidelines: favorable (del 13q as a sole aberration), neutral (normal karyotype, trisomy 12q), and unfavorable (11q and/or 17p deletion);

βs: beta coefficients; SE: standard error of estimated coefficients; FP: feature-specific p-value; LR: likelihood ratio; P: global p-value; Harrell's C: concordance index to evaluate the predictive performance of a survival model. The significant values were shown in boldface (P < 0.05).

**Table S6b: Bivariate models for TTFT in Binet A stage patients included in the validation study**

| Variable | βs | SE | *FP* | HR (95% CI) | LR | *P* | Harrell's C |
| --- | --- | --- | --- | --- | --- | --- | --- |
| *rs1001179*  *(TT genotype)*  *Age* | 0.546  0.001 | 0.291  0.008 | 0.061  0.204 | 1.726 (0.927 – 2.939)  1.011 (0.994 – 1.028) | 4.638 | 0.098 | 0.545 |
| *rs1001179*  *(TT genotype)*  *^b^CD38 positive* | 0.511  0.783 | 0.352  0.238 | 0.146  0.001 | 1.668 (0.778 – 3.145)  2.188 (1.347 – 3.448) | 11.28 | **0.003** | 0.583 |
| *rs1001179*  *(TT genotype)*  *^c^ZAP70 positive* | 0.488  0.485 | 0.352  0.215 | 0.165  0.024 | 1.631 (0.760 – 3.078)  1.625 (1.067 – 2.493) | 6.795 | **0.033** | 0.584 |
| *rs1001179*  *(TT genotype)*  *^d^UM-IGHV* | 0.483  1.325 | 0.292  0.176 | 0.097  <0.0001 | 1.622 (0.871 – 2.764)  3.762 (2.658 – 5.325) | 55.76 | **<0.0001** | 0.657 |
| *rs1001179*  *(TT genotype)*  *^e^mutated TP53* | 0.563  0.876 | 0.292  0.293 | 0.053  0.002 | 1.757 (0.943 – 2.995)  2.403 (1.288 – 4.109) | 10.07 | **0.006** | 0.551 |
| *rs1001179*  *(TT genotype*  *^f^Cytogenetics*  *(unfavorable)* | 0.599  1.080 | 0.294  0.236 | 0.041  <0.0001 | 1.821 (0.974 – 3.118)  2.944 (1.806 – 4.587) | 18.96 | **<0.0001** | 0.572 |

^b^CD38 was determined using a 30% cut-off;

^c^ZAP70 was determined using a 11% cut-off;

^d^*IGHV* sequencing utilized a 2% cut-off to discriminate mutated from unmutated *IGHV*;

^e^*TP53* sequencing utilized a 10% cut-off to discriminate mutated from wild-type *TP53*;

^f^Patients were stratified into major cytogenetic categories, based on NCCN CLL Guidelines: ^3^ favorable (del 13q as a sole aberration), neutral (normal karyotype, trisomy 12q), and unfavorable (11q and/or 17p deletion);

βs: beta coefficients; SE: standard error of estimated coefficients; FP: feature-specific p-value; LR: likelihood ratio; P: global p-value; Harrell's C: concordance index to evaluate the predictive performance of a survival model. The significant values were shown in boldface (P < 0.05).

**Table S7: Association between *CAT* rs1001179 SNP genotypes and clinical parameters in the validation study**

| Parameters | CC/CT | TT | *P* |
| --- | --- | --- | --- |
| *Gender*  Male  Female | 301  197 | 14  18 | *0.0661* |
| *Binet*  Binet A  Binet B/C | 388  88 | 24  5 | *>0.099* |
| *^$^CD38*  Negative  Positive | 200  68 | 15  4 | *0.790* |
| †ZAP70  Negative  Positive | 122  142 | 8  11 | *0.814* |
| *^‡^IGHV*  UM  M | 181  304 | 17  14 | *0.445* |
| *^¥^TP53*  WT  MUT | 440  47 | 29  3 | *>0.099* |
| ^§§^*Cytogenetics*  Favorable/ Neutral  Unfavorable | 387  68 | 28  3 | *0.600* |

^b^CD38 was determined using a 30% cut-off;

^c^ZAP70 was determined using a 11% cut-off;

^d^*IGHV* sequencing utilized a 2% cut-off to discriminate mutated from unmutated *IGHV*; M: mutated; UM: unmutated;

^e^*TP53* sequencing utilized a 10% cut-off to discriminate mutated from wild-type *TP53*; MUT: mutated; WT: wild-type;

^f^Patients were stratified into major cytogenetic categories, based on NCCN CLL Guidelines: ^1^ favorable (del 13q as a sole aberration), neutral (normal karyotype, trisomy 12q), and unfavorable (11q and/or 17p deletion).

**Reference**

1. Hallek M, Shanafelt TD, Eichhorst B. Chronic lymphocytic leukaemia. Lancet 2018;391(10129):1524–1537.
